# Natural Proteasome Inhibitor Celastrol Suppresses Androgen-Independent Prostate Cancer Progression by Modulating Apoptotic Proteins and NF-kappaB

**DOI:** 10.1371/journal.pone.0014153

**Published:** 2010-12-10

**Authors:** Yao Dai, Jeffrey DeSano, Wenhua Tang, Xiaojie Meng, Yang Meng, Ezra Burstein, Theodore S. Lawrence, Liang Xu

**Affiliations:** 1 Department of Radiation Oncology, Division of Radiation and Cancer Biology, University of Michigan Medical School, Ann Arbor, Michigan, United States of America; 2 Departments of Molecular Biosciences, Urology and Radiation Oncology, University of Kansas Cancer Center, University of Kansas, Lawrence, Kansas, United States of America; 3 Departments of Internal Medicine and Molecular Biology, University of Texas Southwestern Medical Center, Dallas, Texas, United States of America; Wayne State University, United States of America

## Abstract

**Background:**

Celastrol is a natural proteasome inhibitor that exhibits promising anti-tumor effects in human malignancies, especially the androgen-independent prostate cancer (AIPC) with constitutive NF-κB activation. Celastrol induces apoptosis by means of proteasome inhibition and suppresses prostate tumor growth. However, the detailed mechanism of action remains elusive. In the current study, we aim to test the hypothesis that celastrol suppresses AIPC progression via inhibiting the constitutive NF-κB activity as well as modulating the Bcl-2 family proteins.

**Methodology/Principal Findings:**

We examined the efficacy of celastrol both *in vitro* and *in vivo*, and evaluated the role of NF-κB in celastrol-mediated AIPC regression. We found that celastrol inhibited cell proliferation in all three AIPC cell lines (PC-3, DU145 and CL1), with IC_50_ in the range of 1–2 µM. Celastrol also suppressed cell migration and invasion. Celastrol significantly induced apoptosis as evidenced by increased sub-G1 population, caspase activation and PARP cleavage. Moreover, celastrol promoted cleavage of the anti-apoptotic protein Mcl-1 and activated the pro-apoptotic protein Noxa. In addition, celastrol rapidly blocked cytosolic IκBα degradation and nuclear translocation of RelA. Likewise, celastrol inhibited the expression of multiple NF-κB target genes that are involved in proliferation, invasion and anti-apoptosis. Celastrol suppressed AIPC tumor progression by inhibiting proliferation, increasing apoptosis and decreasing angiogenesis, in PC-3 xenograft model in nude mouse. Furthermore, increased cellular IκBα and inhibited expression of various NF-κB target genes were observed in tumor tissues.

**Conclusions/Significance:**

Our data suggest that, via targeting the proteasome, celastrol suppresses proliferation, invasion and angiogenesis by inducing the apoptotic machinery and attenuating constitutive NF-κB activity in AIPC both *in vitro* and *in vivo*. Celastrol as an active ingredient of traditional herbal medicine could thus be developed as a new therapeutic agent for hormone-refractory prostate cancer.

## Introduction

The proteasome is a multicatalytic protease complex responsible for the degradation of multiple intracellular proteins that are involved in various cellular events, including DNA repair, cell cycle, survival and apoptosis. For example, p27, a key regulator for G1 to S transition in the cell cycle, is rapidly ubiquitinated and degraded by proteasomes leading to p27's short half-life [1]. Also, most Bcl-2 family proteins are degraded by the ubiquitin-proteasome system (UPS) [2], [3], [4]. Nuclear factor kappaB (NF-κB) is an important pro-survival factor that is pivotally regulated by the proteasome, since it is well known that in the classical NF-κB pathway, IκBα, the sequester of NF-κB, is degraded by the proteasome, therefore liberating NF-κB for activation [5].

Mounting evidence reveals that sustained or constitutive activation of NF-κB contributes to malignant progression and therapeutic resistance in most human cancers [6]. For instance, in prostate cancer, constitutively active NF-κB has been shown to be inversely correlated with androgen receptor status and linked to androgen independence and therapy resistance [7]. The elevated NF-κB surviving signaling pathway in the androgen-independent prostate cancer (AIPC) seems to be correlated with high proteasome activity, as reflected by a faster turnover of IκBα [8], [9]. Based on this evidence, targeting proteasomes might decrease NF-κB activity and thus be a useful strategy in AIPC intervention. In fact, modulation of proteasomal function with specific inhibitors has already been demonstrated as a promising approach to treating human prostate cancer. Bortezomib (Velcade; PS-341), the first to use a proteasome inhibitor in a clinical application, has demonstrated anti-tumor activity when used as a single agent or in combination with conventional therapies in hormone-refractory prostate cancer [10]. In preclinical cancer models, proteasome inhibitors induce prostate cancer apoptosis both *in vitro* and *in vivo* [11], [12], providing substantial evidence that the proteasome inhibitors can be applied as a therapeutic strategy for AIPC.

Celastrol is a natural proteasome inhibitor that has been reported to show anti-proliferative effects and apoptosis in several preclinical cancer models [12], [13]. Mechanistically, NF-κB has been shown to be a key target of celastrol [14]. Recent studies have suggested that celastrol can enhance apoptosis and block either constitutive or induced NF-κB activation with other therapeutic agents, such as tumor necrosis factor [15], temozolomide [16] and gambogic acid [17]. Furthermore, celastrol is proposed to suppress xenografted tumor growth via targeting angiogenesis [18], [19]. In the prostate cancer setting, celastrol alone triggers apoptosis by means of proteasome inhibition and suppresses prostate tumor growth [12]. However, the direct mechanism of action remains elusive. In the current study, we aim to test the hypothesis that celastrol suppresses AIPC progression via inhibiting the constitutive NF-κB activity as well as modulating the Bcl-2 family proteins. We determined the efficacy of celastrol both *in vitro* and *in vivo*, and evaluated the role of NF-κB in celastrol-mediated AIPC regression.

## Materials and Methods

### Reagents and Cell Culture

Celastrol (98% purity) was purchased from Gaia Chemical (Gaylordsville, CT). The powder was resolved in dimethyl sulfoxide (DMSO) and stored as aliquots (20 mM) at −70°C. MG-132 was purchased from BIOMOL (Plymouth Meeting, PA). Protease inhibitor cocktail was provided by Roche (Indianapolis, IN). Other chemicals were purchased from Sigma unless otherwise indicated. Human prostate cancer cell lines PC-3, DU145 and LNCaP were purchased from American Type Culture Collection. The androgen-independent prostate cancer cell line CL1, derived from the androgen-dependent LNCaP cell line [20], was kindly provided by Dr. Arie Belldegrun (University of California, Los Angeles, CA). Cells were maintained in RPMI-1640 (PC-3) or DMEM (DU145, LNCaP and CL1) supplemented with 10% FBS, 100 U/ml penicillin, and 100 µg/ml streptomycin, and incubated in a 5% CO_2_ humidified incubator at 37°C.

### Cell Proliferation and Cell Death

Cells were seeded in a 96-well plate and treated with celastrol in triplicate. After 4 days of incubation, viable cells were identified using a cell counting kit with WST-8 (Dojindo, Rockville, MD) and absorbance was tested at 450 nm colorimetrically. Cell viability (%) was the ratio of absorbance of treated sample to untreated control [21],[22]. Alternatively, cells were seeded in a 24-well plate at a density of 2×10^5^ cells/well and treated with celastrol in duplicate wells. Attached cells were harvested and counted using a Coulter cell counter (Fullerton, CA) every 24 h for 4 days. Cell death was tested by trypan blue exclusion for both floating and attached cells [21], [23]. Data were plotted and analyzed by GraphPad Prism 5.0 (San Diego, CA). Fifty percent inhibitory concentrations (IC_50_) were calculated using a sigmoidal dose-response nonlinear regression analysis (Prism 5.0) [21], [22], [24].

### Cell Migration

Cell migration was determined by a “wound-healing” assay [25], [26]. Cells were seeded in a 6-well plate and grew for 48 h to reach confluence. Gaps were created artificially by scratching the cell monolayer. Cell migration was photographed 6 h, 24 h and 48 h after each scratch. Cells migrating into the denuded area were scored from four random fields.

### Cell Invasion

Invasion was tested using Transwell (8 µm pore) pre-loaded with Matrigel (BD Biosciences, San Diego, CA). Cells were incubated with or without celastrol (1 µM) in a serum-free medium and loaded onto the top chamber (2×10^4^/insert). The complete medium (10% FBS) was added to the lower chamber to generate a chemotractant. Twenty-four hours after cell seeding, the inserts were wiped with a cotton swab to remove the MatriGel, and stained with crystal violet (0.1%). Invading cells on the underside of the filter were scored from eight random fields.

### Cell Cycle Analysis

Cells were fixed in 70% ethanol at 4°C overnight, and then treated with propidium iodide (PI, 50 µg/ml) and RNase A (1 µg/ml) for 30 min. Samples were analyzed by flow cytometry (FACScalibur, BD Biosciences). The sub-G1 population was scored from hypodiploid DNA content [24], [27]. Data were analyzed using WinMDI 2.8 software (Purdue University Cytometry Laboratory).

### Caspase Activation

Cells were homogenized in a lysis buffer (BioVision, Mountain View, CA), and whole cell lysates (40 µg) were incubated with 20 µM of fluorogenic substrate LEHD-AFC or DEVD-AFC in a reaction buffer (BioVision) containing 5 mM DTT at 37°C for 1 h. Proteolytic release of AFC was monitored at λex = 405 nm and λem = 500 nm using a fluorescence microplate reader (BMG LABTECH, Cary, NC). Activity is expressed as “fold increase”, and calculated as the ratio of fluorescence signal in treated samples to untreated control cells.

### Western Blot

Whole cell lysates of cells or tumor tissues were made and Western blot experiments were carried out as we described previously [26]. Antibodies against poly(ADP-ribose) polymerase (PARP) (F-2), Mcl-1 (S-19), Bcl-2 (C-2), ubiquitin (P4D1), caspase-3 (H-277), IκBα (C-15), RelA (F-6), tubulin (4G1) and GAPDH (L-20) were obtained from Santa Cruz Biotechnology (Santa Cruz, CA). Anti-Noxa antibody was purchased from Calbiochem (Gibbstown, NJ). Anti-β-actin (AC-74) antibody was obtained from Sigma (St. Louis, MO). Band density was quantified by TotalLab software (Durham, NC).

### Subcellular Fractionation

Cytosolic and nuclear fractions were prepared as previously described [24], [25]. Briefly, cytosolic extract was obtained by homogenizing cells in a hypotonic buffer [10 mM HEPES, 5 mM KCl, 1.5 mM MgCl_2_ and 1 mM dithiothreitol (DTT) with protease inhibitor cocktail], followed by the addition of Igepal CA-630 (0.5%). The pellets were resolved in a hypertonic buffer (20 mM HEPES, 50 mM KCl, 300 mM NaCl, 1 mM PMSF, 1 mM DTT, and protease inhibitors) to obtain nuclear extracts. Subcellular proteins were quantified by Bradford assay before Western blot.

### Quantitative Real-time PCR (qPCR)

qPCR was performed to determine NF-κB target gene expression [28]. Total RNA was extracted using TRIzol reagent (Invitrogen, Carlsbad, CA) according to the manufacturer's instructions. cDNA was obtained by reverse transcription with 1 µg of total RNA using a TaqMan Reverse Transcription Kit (Applied BioSystems). SYBR Green was used for quantitative PCR. Sequences of gene primers are listed in Table S1. Reactions with TaqMan PCR Master Mix (Applied BioSystems) were performed on the Mastercycler *realplex* ^2^ S system (Eppendorf, Westbury, NY). mRNA levels of target genes were normalized to GAPDH using the formula: [2∧−(C_T_ target−C_T_ Actin)]×100%, where C_T_ is the threshold cycle [27]. mRNA expression is expressed as “fold increase” and was calculated by dividing the normalized target gene expression of the treated sample with untreated control cells.

### Animal Study

Female athymic nu/nu mice (5 weeks) were inoculated subcutaneously (s.c.) with PC-3 cells (3×10^6^) on both sides of the lower back. When tumors grew to 100 mm^3^, the mice were randomized and treated daily via oral gavage (p.o.) with vehicle control [12] or celastrol at a dose of 1 mg/kg with 5 days per week for 3 weeks. Tumor size was measured on the last dosing of celastrol (day 18). Tumor volume was calculated as (length×width^2^)/2. Tumor samples were fixed in 4% paraformaldehyde and embedded by paraffin. Immunohistochemical analysis was performed by Ki67, terminal deoxynucleotidyl transferase biotin-dUTP nick end labeling (TUNEL), and CD31 staining for the detection of proliferation, apoptosis and angiogenesis, respectively, as we previously described [22], [23], [26]. All animal experiments were carried out according to protocols approved by University of Michigan Guidelines for Use and Care of Animals.

### Statistical Analysis

Two-tailed Student's *t*-test was employed for analyzing both *in vitro* and *in vivo* data. All analysis was performed by GraphPad Prism 5.0. A threshold of *P*<0.05 was defined as statistically significant.

## Results

### Celastrol Inhibits Prostate Cancer Cell Proliferation

**Figure 1:**
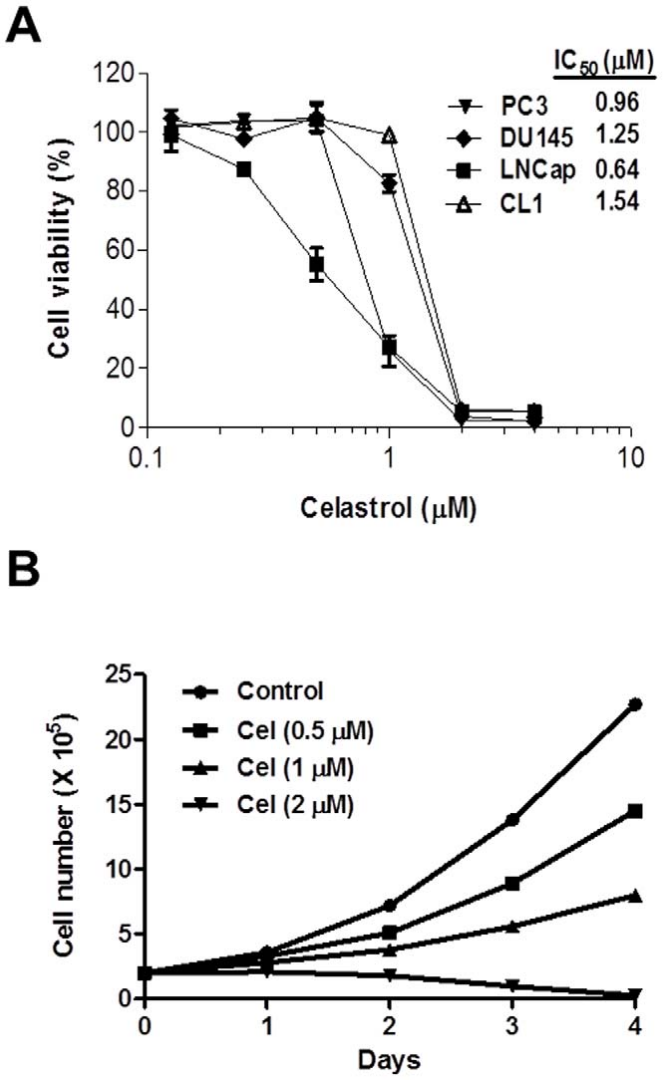
Celastrol inhibited prostate cancer cell proliferation. (**A**): cells were treated with various doses of celastrol and cell viability was determined by MTT assay. IC_50_ was shown as calculated by Prism 5.0. Data are mean ± SD (n = 3). (**B**): PC-3 cells were treated with different doses of celastrol (Cel). Attached cells were harvested and counted every day for 4 days. Data shown are mean ± SD (n = 6).

Celastrol has been reported to show anti-tumor activity in human prostate cancer cells [12]. In our study, we confirmed that celastrol reduced cell viability in both androgen-dependent (LNCaP) and androgen-independent (PC-3, DU145, CL1) prostate cancer cells, with an IC_50_ of cytotoxicity less than 2 µM (Fig. 1A). For PC-3 cells, celastrol inhibited proliferation dose-dependently (Fig. 1B), with 65% growth inhibition at the IC_50_ dose (∼1 µM) on day 3. At 2 µM, celastrol completely inhibited cell growth in the cell lines tested. These data demonstrate that celastrol consistently decreases AIPC cell proliferation and viability at the concentrations <2 µM.

### Celastrol Suppresses Migration and Invasion

**Figure 2:**
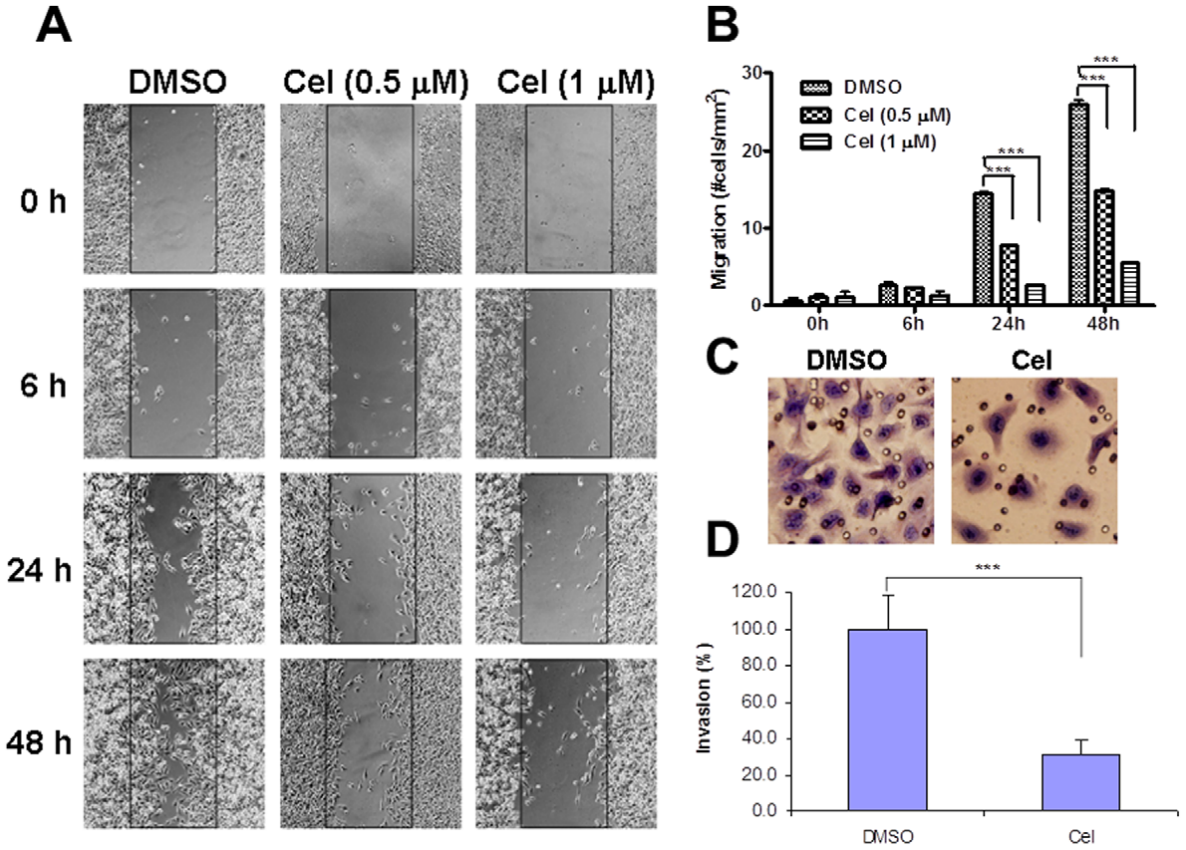
Celastrol inhibited cell migration and invasion in AIPC cells. (**A**): PC-3 monolayer cells were scratched and treated with celastrol at 0.5 and 1 µM. “Wound-healing” was tested at 6 h, 24 h and 48 h post-scratch. Original magnification, ×100. (**B**): quantification of cell migration in A from 4 random fields in duplicate samples. *Columns*, mean; *bars*, SD (n = 8). ***, *P*<0.001. (**C**): DU145 cells treated with or without 1 µM of celastrol were seeded into Matrigel-coated transwell inserts (2×10^4^/insert) for 24 h. Cells on the underside of the filter were stained. Original magnification, ×200. (**D**): invading cells in (**C**) were scored from 8 random fields. *Columns*, mean; *bars*, SD (n = 8). ***, *P*<0.001.

As a higher concentration of celastrol was cytotoxic (Fig. 1), we evaluated the effect of celastrol on cell migration and invasion using non-toxic doses (<1 µM). Celastrol inhibited PC-3 cell migration in both a dose- and time-dependent manner (Fig. 2A). With concentrations (0.5 µM) that minimally affected proliferation, celastrol prevented cell motility 24 h post-treatment (*P*<0.001, Fig. 2B). Moreover, DU145 cell invasion was inhibited to 70% by 1 µM of celastrol (*P*<0.001, Fig. 2C and D). These data suggest that celastrol suppresses AIPC cell migration and invasion at subcytotoxic concentrations.

### Celastrol Induces Apoptosis and Modulates Mcl-1

**Figure 3:**
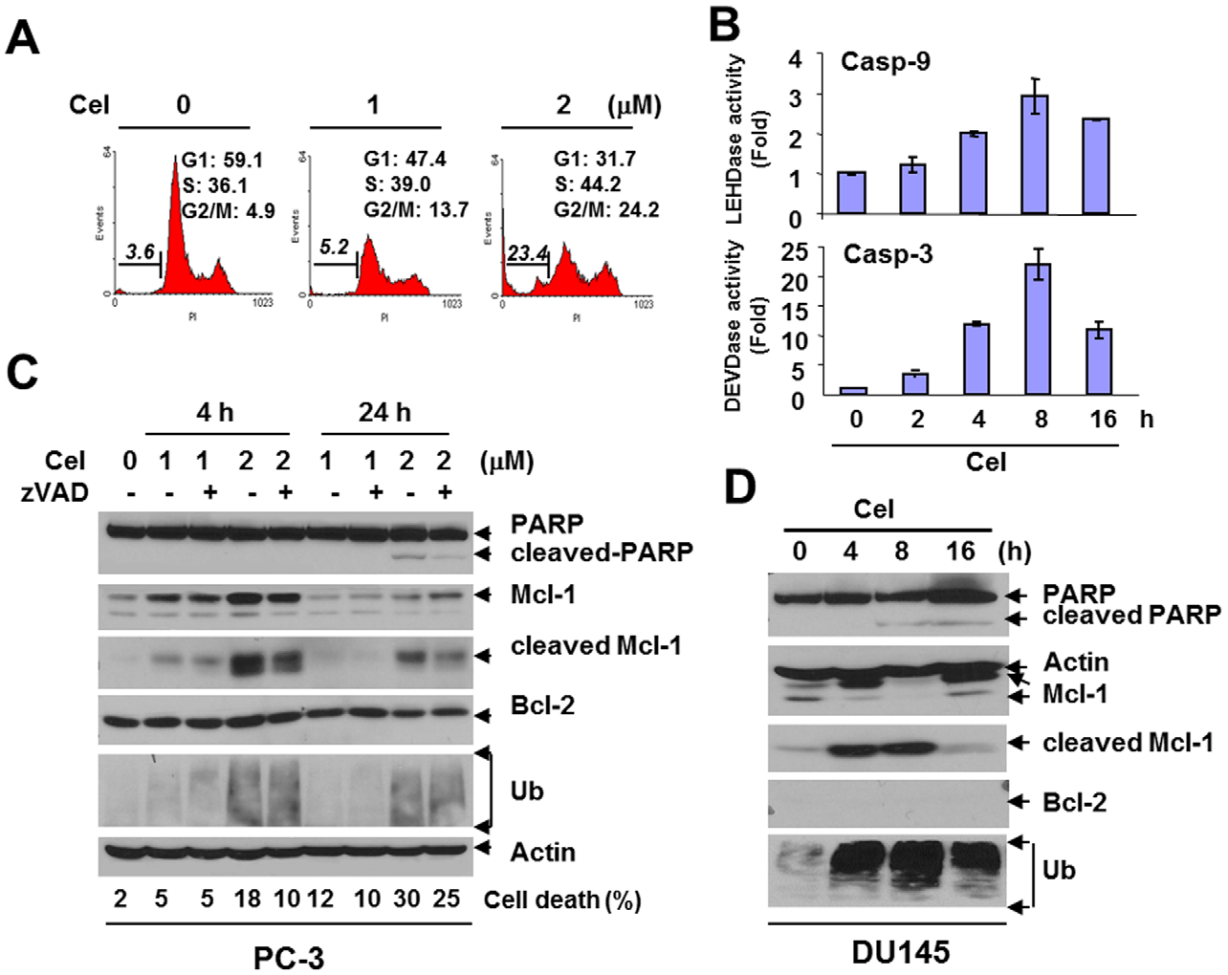
Celastrol induced apoptosis in AIPC cells. (**A**): PC-3 cells were treated with celastrol for 24 h and cell cycle was tested. Cell population in each cell cycle phase was numerically depicted. Data represent one of three independent experiments. (**B**): DU145 cells were treated with celastrol (2 µM) for desired time points. Enzymatic activity of caspase-9 and caspase-3 was determined fluorometrically. *Columns*, mean; *bars*, SD (n = 3). (**C**): PC-3 cells were treated with celastrol with or without 1 h pretreatment of the pan-capsase inhibitor zVAD (2 µM) for Western blot analysis. Actin was used as a loading control. Cell death was detected by trypan blue exclusion. (**D**): DU145 cells were treated with celastrol (2 µM) for Western blot. Actin is a loading control.

We next investigated whether apoptosis is involved in celastrol-mediated cytotoxicity. Celastrol indeed induced chromatin condensation and DNA fragmentation in AIPC cells. PC-3 treated with 2 µM of celastrol for 24 h produced an 8-fold increase in sub-G_1_ population (Fig. 3A). In addition, celastrol induced G_2_/M arrest (Fig. 3A) that was consistent with an earlier report [29], further demonstrating celastrol's effect on cell cycle arrest. Celastrol also dramatically enhanced enzymatic activity of both caspase-9 and caspase-3 in DU145, with the strongest activation (2.9±0.4-fold for caspase-9 and 22.3±3.5-fold for caspase-3, respectively, *P*<0.001) at 8 h post-treatment (Fig. 3B), indicating the amplification of the caspase cascade in the intrinsic apoptotic pathway. Celastrol induced PARP cleavage that could be reversed by the pan-caspase inhibitor zVAD, demonstrating the apoptosis is caspase-dependent (Fig. 3C). Interestingly, celastrol was shown to simultaneously accumulate and cleave the anti-apoptotic protein Mcl-1 at an early (4 h) rather than a late stage (24 h) in PC-3 (Fig. 3C). Such observation was consistent with the expression of intracellular ubiquitin (Fig. 3C), suggesting a rapid and complex regulation of Mcl-1 by the proteasome-inhibitor. In DU145, a similar phenomenon was observed. Mcl-1 cleavage that occurred in the early stage (4–8 h) (Fig. 3D) was correlated with caspase activation (Fig. 3B). Furthermore, induction of cleaved Mcl-1 was reversed by zVAD (Fig. 3C), indicating that such cleavage is mediated by caspases. In contrast, little change was observed for Bcl-2 (Fig. 3C), Bcl-xL or XIAP (data not shown), suggesting that among the anti-apoptotic Bcl-2 family proteins, Mcl-1 appears to be a major target of celastrol in the cell lines tested.

### Celastrol Induces Apoptosis by Activating Noxa

**Figure 4:**
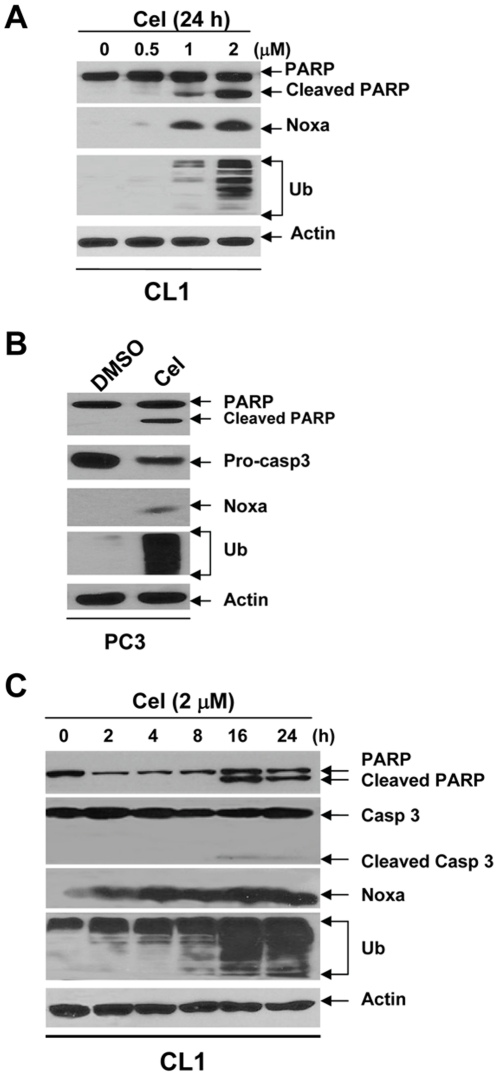
Celastrol induced Noxa expression in AIPC cells. (**A**): CL1 cells were treated with celastrol for 16 h. (**B**): PC-3 cells were treated with celastrol (2 µM) for indicated time points. (**C**): CL1 cells were treated with celastrol (2 µM) for 16 h. Whole cell lysates were analyzed by Western blot, with Actin as a loading control.

It has been shown that Noxa, a BH3-only pro-apoptotic protein, can be significantly induced by bortezomib [30]. In the current study, we investigated whether Noxa might also be activated by celastrol. Indeed, celastrol induced Noxa expression that was consistent with caspase-3 activation, PARP cleavage and ubiquitin accumulation dose-dependently in CL1 cells (Fig. 4A) and PC-3 cells (Fig. 4B). Furthermore, Noxa induction occurred as early as 2 h post-treatment, much more quickly than caspase-3 activation and PARP cleavage in CL1 (Fig. 4C). Together, these data suggest that Noxa is strongly and rapidly activated by celastrol, which may be responsible for the pro-apoptotic effect in AIPC cells.

### Celastrol Suppresses the Constitutive NF-κB Activity

**Figure 5:**
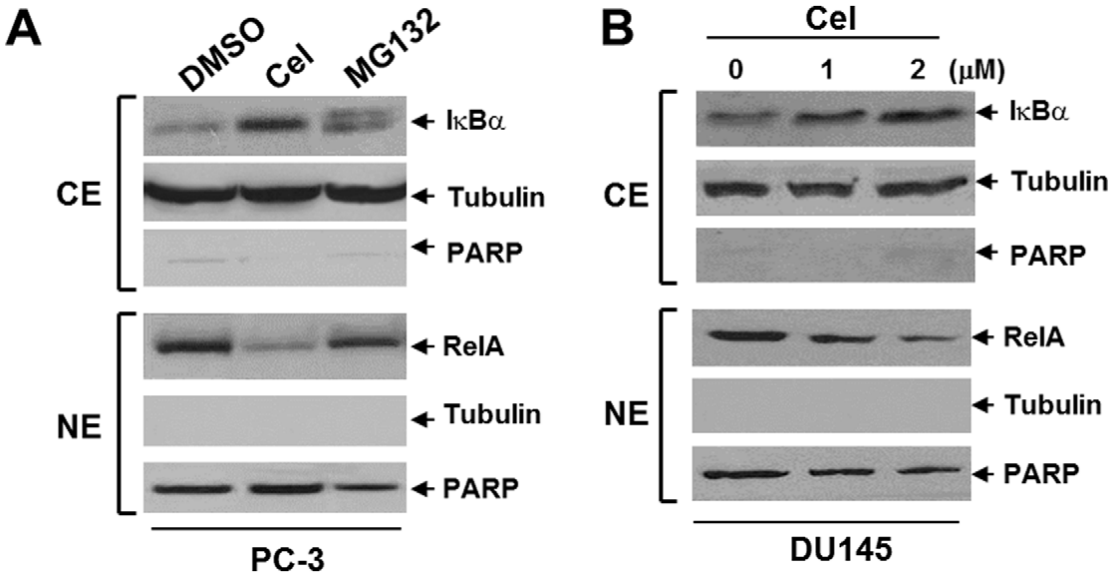
Celastrol altered subcellular distribution of NF-κB in AIPC cells. (**A**): PC-3 cells were treated with celastrol (2 µM) or MG132 (10 µM) for 30 min. (**B**): DU145 cells were treated with celastrol (1 and 2 µM) for 30 min. For both (**A**) and (**B**), cells were processed for subcellular fractionation. Cytosolic extract (CE) was probed for IκBα and nuclear extract (NE) was probed for RelA. Tubulin and PARP are used as marker and loading control for CE and NE, respectively.

It is well known that proteasome inhibitors, including celastrol, can block NF-κB activity in various human cancers [15]. In our study, we tested whether a similar result could be obtained in AIPC cells. In PC-3 cells, celastrol blocked cytosolic IκBα degradation, as well as nuclear translocation of NF-κB protein RelA/p65, showing effects that exceeded MG132, a widely used proteasome inhibitor that is shown to block NF-κB signaling [31] (Fig. 5A). Similarly, celastrol dose-dependently caused subcellular redistribution of NF-κB in DU145 cells (Fig. 5B). Moreover, much like MG132, celastrol inhibited the expression of various NF-κB target genes that are involved in multiple steps of tumor progression, including proliferation (CXCL1, c-Myc and Cyclin-D1), adhesion (ICAM-1), migration (CXCL1), invasion (MMP-9), and anti-apoptosis (BIRC2/4/5, Bcl-2 and Bcl-xL) (**Table. 1**). The inhibitory effect of NF-κB by celastrol was further confirmed by the luciferase reporter assay (data not shown). These data demonstrate that celastrol may suppress AIPC progression by impeding constitutive NF-κB activity.

**Table 1:** Summary of NF-κB target gene expression by proteasome inhibitors.

Gene	DMSO	Celastrol (1 µM)			Celastrol (2 µM)			MG132 (1 µM)
		1 h	2 h	4 h	1 h	2 h	4 h	1 h
**TNF**	1.01±0.11	0.78±0.21	0.73±0.01	0.26±0.03	0.36±0.10	0.30±0.09	0.29±0.13	0.43±0.13
**CXCL1**	1.00±0.03	0.56±0.04	0.48±0.06	0.22±0.01	0.43±0.03	0.38±0.04	0.37±0.01	0.43±0.03
**c-Myc**	1.00±0.03	0.59±0.05	0.58±0.04	0.29±0.02	0.47±0.01	0.62±0.06	0.48±0.03	0.45±0.02
**Cyclin D1**	1.00±0.04	0.52±0.03	0.78±0.01	0.61±0.03	0.56±0.03	0.68±0.01	0.58±0.02	0.41±0.01
**ICAM-1**	1.01±0.11	0.32±0.03	0.86±0.08	0.58±0.02	0.25±0.02	0.40±0.04	0.35±0.07	0.31±0.06
**MMP9**	1.01±0.10	0.58±0.04	0.38±0.06	0.37±0.05	0.36±0.18	0.38±0.03	0.47±0.17	0.22±0.02
**BIRC2**	1.01±0.07	0.68±0.08	1.26±0.11	0.70±0.05	0.65±0.05	0.68±0.07	0.84±0.05	0.48±0.00
**BIRC4**	1.00±0.04	0.43±0.03	0.45±0.01	0.28±0.02	0.38±0.01	0.36±0.01	0.30±0.01	0.31±0.01
**BIRC5**	1.00±0.01	0.47±0.03	0.50±0.02	0.46±0.00	0.49±0.03	0.49±0.02	0.41±0.01	0.34±0.00
**Bcl-2**	1.00±0.06	0.50±0.07	0.56±0.06	0.29±0.03	0.36±0.01	0.59±0.01	0.31±0.02	0.32±0.02
**Bcl-xL**	1.01±0.08	0.66±0.04	0.66±0.04	0.47±0.02	0.50±0.01	0.65±0.02	0.48±0.02	0.38±0.00

Note: Real-time PCR analysis of NF-κB target gene expression in PC3 cells. MG132 was used as a positive control for proteasome inhibition. Data are presented as mean ± SEM (*n* = 3).

### Anti-tumor Effect of Celastrol Involves NF-κB Attenuation in PC-3 Xenografts

**Figure 6:**
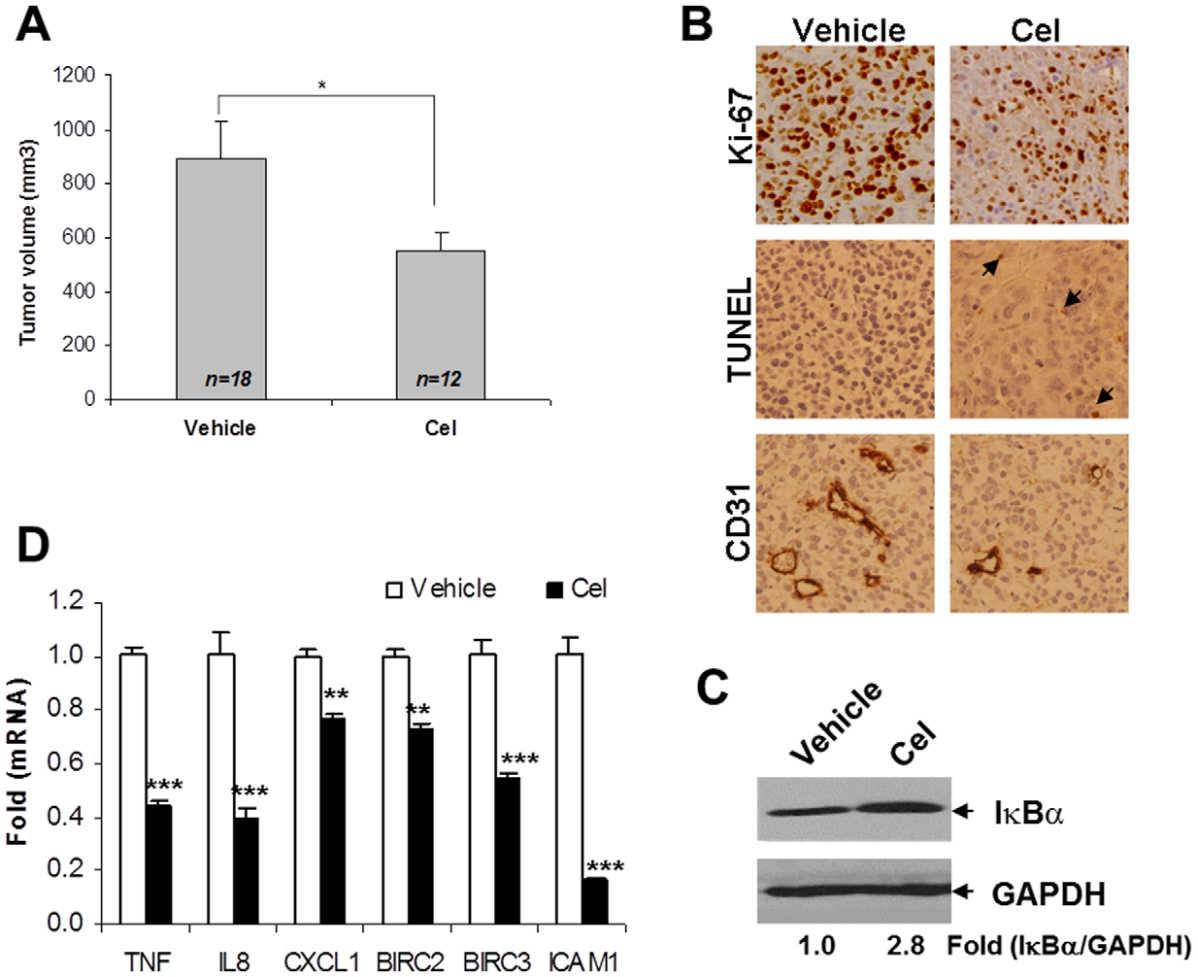
Celastrol suppressed PC-3 tumor progression and attenuated NF-κB activity. (**A**): tumor volume was measured after celastrol treatment. The number of mice in each group was shown. *, *P*<0.05. (**B**): tumor sections were processed by anti-Ki67, TUNEL and anti-mouse CD31 staining. Original magnification, ×400. (**C**): whole cell lysates (50 µg) of tumor tissues were probed with anti-IκBα antibody. GAPDH was used as a loading control. Expression fold was the ratio of IκBα expression in the celastrol group to vehicle control. (**D**): qPCR analysis of NF-κB target gene expression in tumors. Total RNA from tumor tissues was extracted for reverse transcription. Gene primers were mixed with cDNA and SyberGreen for qPCR. *Columns*, mean; *bars*, SD (n = 3). **, *P*<0.01; ***, *P*<0.001.

To determine whether NF-κB suppression would contribute to tumor regression *in vivo*, we evaluated celastrol efficacy in the established PC-3 xenografted model as decribed previously [26]. Treatment of celastrol (1 mg/kg) for 3 weeks inhibited PC-3 tumor growth (*P*<0.05) (Fig. 6A), with a minimal systemic toxicity [26]. Histological analysis showed that celastrol reduced Ki67-positive cells and CD31-positive microvessels, and increased TUNEL-positive cells (Fig. 6B), suggesting that celastrol decreases proliferation and angiogenesis, and induces apoptosis in tumor tissues. Furthermore, total IκBα was shown to have accumulated after treatment (Fig. 6C). Also, expression of NF-κB target genes that are involved in proliferation (CXCL1), angiogenesis (IL-8) and anti-apoptosis (BIRC2 and BIRC3) was inhibited by celastrol in tumors (*P*<0.01 for CXCL1 and BIRC2; *P*<0.001 for other genes) (Fig. 6D). These data indicate that attenuation of NF-κB correlates with PC-3 tumor regression by celastrol *in vivo*.

## Discussion

**Figure 7:**
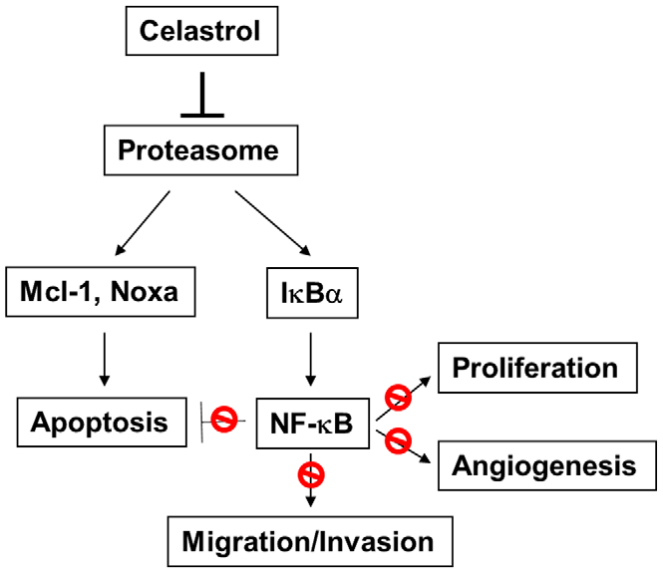
Diagram of celastrol effects on AIPC progression. Celastrol blocks proteasome function, resulting in Mcl-1 turnover and noxa induction that triggers apoptosis. Simultaneously, celastrol prevents IκBα degradation, leading to NF-κB suppression. Overall, celastrol disrupts AIPC cell proliferation, migration, invasion and angiogenesis, and induces apoptosis by significantly impeding constitutive NF-κB activity.

In this study, we have found that celastrol, a natural proteasome inhibitor, suppresses AIPC progression by modulating two pathways. First, celastrol induces caspase-dependent apoptosis by regulating anti-apoptotic protein Mcl-1 and activating pro-apoptotic protein Noxa. Second, by preventing IκBα degradation, celastrol attenuates constitutive NF-κB activity and thus abrogates the inhibitory effect of NF-κB on apoptosis as well as the promotion of proliferation, angiogenesis, migration and invasion (Fig. 7). Overall, these data elucidate the potential efficacy and mechanism of celastrol in treating AIPC via targeting the proteasome.

It has been shown that dysregulated proteasome function is correlated with tumor progression and therapy resistance in human cancers [32]. Hyperactivation of NF-κB correlates with the phenotype of androgen independence and therapy resistance in AIPC [7], [33]. In PC-3 cells, high constitutive NF-κB activity is, at least in part, the result of a high level of proteasome activity [34]. In the current study, we report that celastrol not only potently inhibits PC-3 growth, migration, invasion, and angiogenesis, but also induces apoptosis that is associated with NF-κB attenuation both *in vitro* and *in vivo*. This finding suggests that in AIPC, suppressing NF-κB by targeting proteasome is applicable in disrupting tumor progression, including primary growth and metastasis. Although more evidence is needed to delineate the role of NF-κB in celastrol-mediated tumor regression, our current study reveals that celastrol inhibits multiple NF-κB-driven gene expression that is involved in AIPC growth and metastasis. Our findings, consistent with others [35], [36], demonstrate that NF-κB is a pivotal mediator of AIPC progression, and celastrol, functioning as an active natural proteasome inhibitor, can significantly impair AIPC progression.

Mcl-1 is an anti-apoptotic protein in the Bcl-2 family that is rapidly degraded by the proteasome and, therefore has a short half-life (<3 h) [37]. An increasing number of studies propose that proteasome inhibitors are able to reverse Mcl-1 function via cleaving its original molecule by caspases to generate a short form (26∼28 kDa) that is pro-apoptotic regardless of the direct stabilization of the full length of Mcl-1 [38], [39]. Such rapid turnover of Mcl-1 highlights the quick response by cancer cells once they encounter proteasome stress, switching the phenotype from cell survival to programmed cell death. Consistent with bortezomib [38], [40], we find that in AIPC, celastrol also significantly regulates Mcl-1 at an early stage by paradoxically accumulating the anti-apoptotic original form, while also generating the pro-apoptotic cleaved form. As cells undergo apoptosis eventually, it is reasonable to postulate that cleaved Mcl-1 may be more important in controlling cellular behavior than previously reported [41]. This is because Mcl-1 cleavage occurs along with caspase activation and before PARP cleavage, while increased intact Mcl-1 is only a transient event in response to proteasome inhibition. Induction of cleaved Mcl-1 can be partially attenuated by the caspase inhibitor, suggesting that such an induction is caspase-dependent. Interestingly, cleaved Mcl-1 levels decrease at a later stage, which is consistent with intact Mcl-1. This suggests that even after cleavage by caspases, the Mcl-1 fragment is still regulated by proteasomes. Taken together, these observations indicate that in AIPC cells, Mcl-1 is a key target of celastrol that exhibits a complex response by proteasome inhibition.

Much like Mcl-1, Noxa is another Bcl-2 family protein that is strongly increased by proteasome inhibition in different cancers, including melanoma [30], [42] and multiple myeloma [39], [43]. However, unlike Mcl-1, Noxa is not a direct substrate of proteasome [44]. Instead, Noxa mRNA is transcriptionally enhanced by a proteasome inhibitor [44], [45]. Noxa is a BH3-only pro-apoptotic protein functioning in mitochondrial apoptosis [4], [22]. Upon stress, Noxa can be activated in a p53-dependent manner and interact with anti-apoptotic proteins, thus abolishing their negative effect on apoptosis [46]. Our results suggest that Noxa expression occurs even before activation of the initiator caspase-9, indicating that it is an early mediator of celastrol-induced apoptosis. Intriguingly, none of the three AIPC cell lines has functional p53 (PC-3 and CL1: p53 null; DU145: p53 mutant). Thus the Noxa induction by celastrol is p53-independent. How Noxa is positively regulated in a p53-deficient scenario is unclear. Nevertheless, as Noxa induction correlates with Mcl-1 accumulation, and since the Mcl-1/Noxa complex is reported to be increased by bortezomib [39], current data indicate that the potential function of the induced Noxa may be interacting with accumulated Mcl-1 and neutralizing its anti-apoptotic effect. Together, these data reveal that both Mcl-1 and Noxa exhibit rapid and multi-faceted turnover events upon proteasome inhibition, and celastrol's coordination of these two Bcl-2 family members will lead to apoptosis through the initiation of the caspase cascade in AIPC.

In summary, our data outline the potent anti-tumor effects of the traditional natural proteasome inhibitor celastrol on androgen-independent prostate cancer. The dual role of celastrol, modulating both apoptotic proteins and NF-κB, warrants its consideration as a potential therapeutic candidate in treating hormone-refractory prostate cancer patients with constitutively active NF-κB.

## Supporting Information

Supplemental Table S1. Primers for real-time PCR (human).
(0.01 MB PDF)
