# Supplementary material for: Natural Proteasome Inhibitor Celastrol Suppresses Androgen-Independent Prostate Cancer Progression by Modulating Apoptotic Proteins and NF-kappaB
Source: PLoS One. 2010 Dec 10;5(12):e14153. doi: 10.1371/journal.pone.0014153 (PMC3000808; doi:10.1371/journal.pone.0014153)
Supplement: Supplemental Table S1. — Primers for real-time PCR (human). (0.01 MB PDF) [file pone.0014153.s001.pdf]

Supplemental Table S1.

Table S1. Primers for real-time PCR (human)

Gene	Length	Primer sequences (5' to 3')
TNF	107 bp	Forward: CCAGGGACCTCTCTCTAATCAGC<br>Reverse: CTCAGCTTGAGGGTTTGCTACAA
IL8	75 bp	Forward: CGTGGCTCTCTTGGCAGC<br>Reverse: TCTTTAGCACTCCTTGGCAAAAC
CXCL1	112 bp	Forward: CTTGCCTCAATCCTGCATC<br>Reverse: CCTTCTGGTCAGTTGGATTTG
c-Myc	72 bp	Forward: GCCACGTCTCCACACATCAG<br>Reverse: TCTTGGCAGCAGGATAGTCCTT
cyclin D1	75 bp	Forward: CCGTCCATGCGGAAGATC<br>Reverse: GAAGACCTCCTCCTCGCACT
ICAM-1	111 bp	Forward: CAGGGGACCGTGGTCTGTT<br>Reverse: CATAGGTGACTGTGGGGTTCAA
MMP-9	111 bp	Forward: GAGGCGCTCATGTACCCTATGT<br>Reverse: CCGTGGCTCAGGTTCAGG
BIRC2	101 bp	Forward: TGGATTTGTTGTTCTTTCAGAAAG<br>Reverse: TTCGGCACCATAACTCTGATGA
BIRC3	101 bp	Forward: AACAAAACACCAGGGACACATTTC<br>Reverse: AAACACCATGTCCCTAAAATGTCATT
BIRC5	65 bp	Forward: TGCCTGGCAGCCCTTTC<br>Reverse: CCTCCAAGAAGGGCCAGTTC
Bcl-2	76 bp	Forward: CATGCTGGGGCCGTACAG<br>Reverse: GAACCGGCACCTGCACAC
Bcl-xL	142 bp	Forward: ATGGGGTAAACTGGGGTCG<br>Reverse: GGCTCTAGGTGGTCATTCAGG
GAPDH	73 bp	Forward: TCCATGACAACTTTGGTATCGTG<br>Reverse: ACAGTCTTCTGGGTGGCAGTG
